# Supplementary material for: Gelatin-Based Biofilms with FexOy-NPs Incorporated for Antioxidant and Antimicrobial Applications
Source: Materials (Basel). 2022 Mar 7;15(5):1966. doi: 10.3390/ma15051966 (PMC8912019; doi:10.3390/ma15051966)
Supplement: Supplementary file 1 [file materials-15-01966-s001.zip › materials-1602553-supplementary.pdf]

materials

SUPLEMENTARY MATERIALS

Gelatin-Based Biofilms with Fe_xO_y-NPs Incorporated for Antioxidant and Antimicrobial Applications

Johar Amin Ahmed Abdullah [1,*], Mercedes Jiménez-Rosado [1], Antonio Guerrero [1] and Alberto Romero [2]

[1] Departamento de Ingeniería Química, Escuela Politécnica Superior, Universidad de Sevilla, 41011 Sevilla, Spain; mjimenez42@us.es (M.J.-R.); aguerrero@us.es (A.G.)
[2] Departamento de Ingeniería Química, Facultad de Física, Universidad de Sevilla, 41012 Sevilla, Spain; alromero@us.es
* Correspondence: jabdullah@us.es; Tel.: +34-95-455-7179

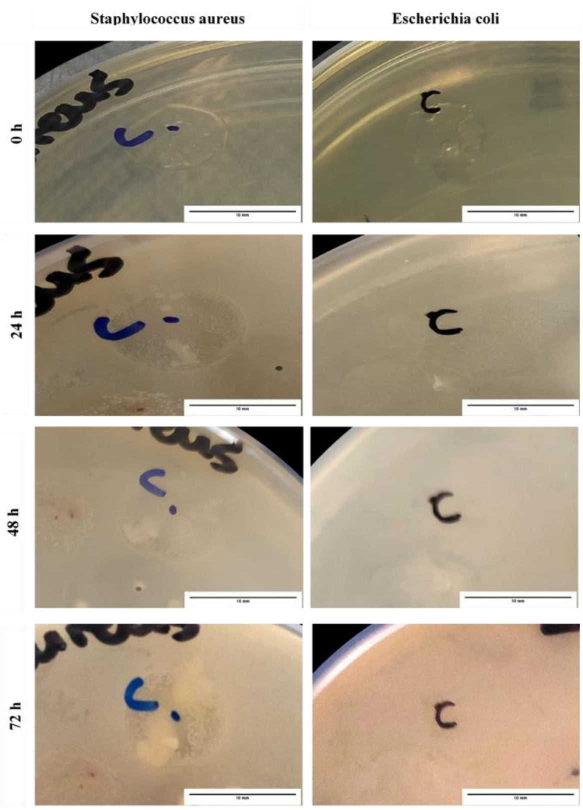

Figure S1: Image of inhibition area over time of Neat gelatin-based biofilm without Fe_xO_y-NPs incorporated.

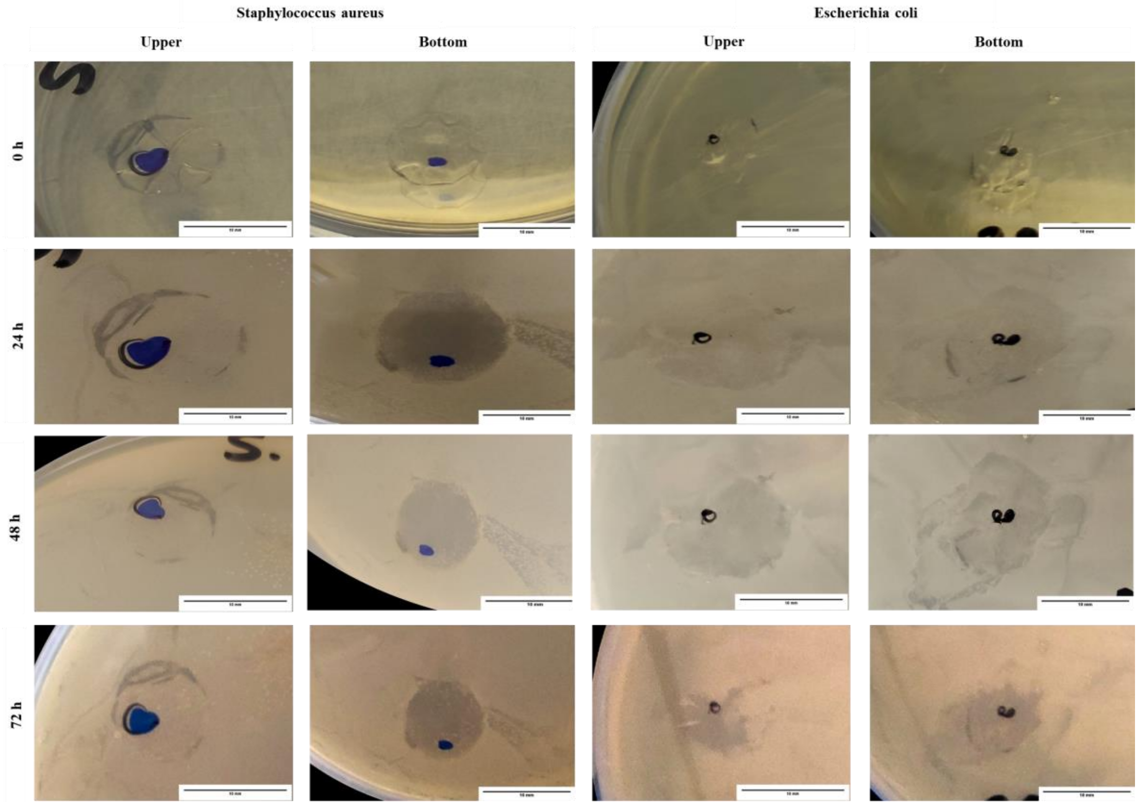

Figure S2: Image of inhibition area over time of gelatin-based biofilm with 1.0 % GS Fe_xO_y-NPs incorporated.

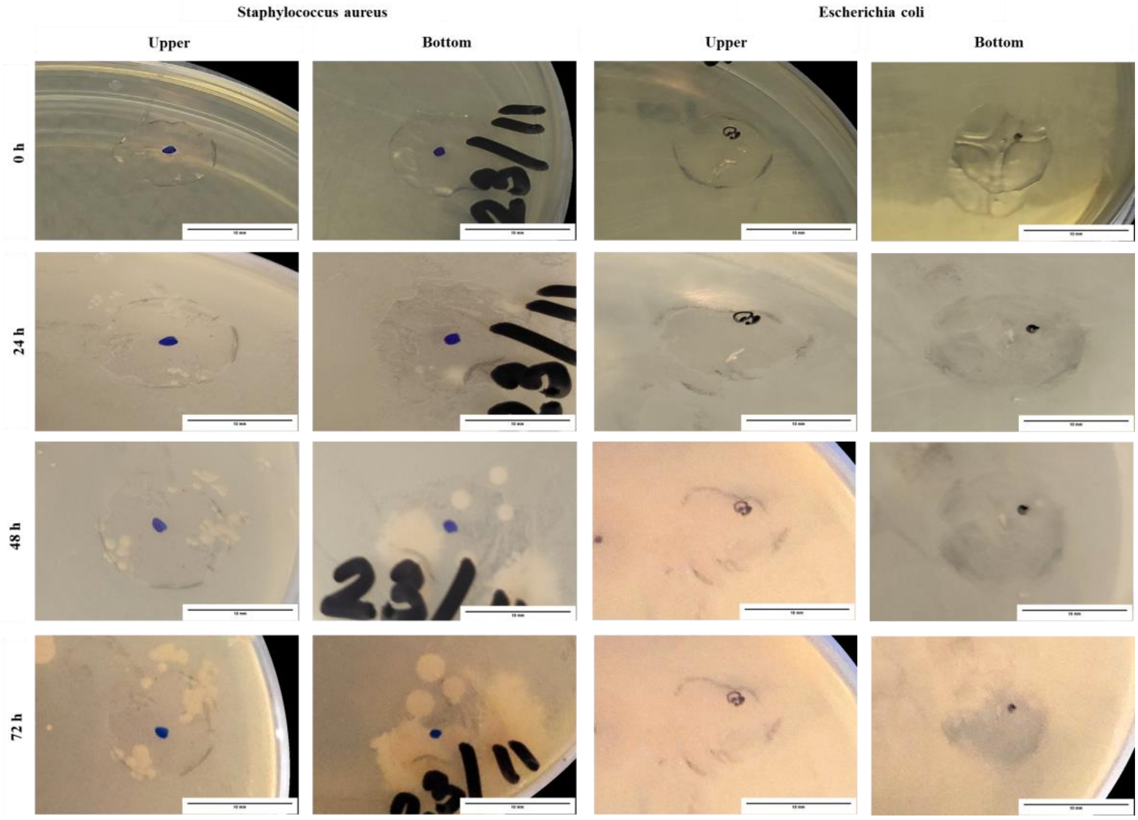

Figure S3: Image of inhibition area over time of gelatin-based biofilm with 1.0 % CS Fe_xO_y-NPs incorporated.
